# Prediction Model for Sciatic Nerve Procedures: A Cross-Sectional Study

**DOI:** 10.3390/jcm13247851

**Published:** 2024-12-23

**Authors:** Isabel Minguez-Esteban, Ángel González-de-la-Flor, Jorge Hugo Villafañe, Juan Antonio Valera-Calero, Gustavo Plaza-Manzano, Pedro Belón-Pérez, Carlos Romero-Morales

**Affiliations:** 1 Department of Physiotherapy, Faculty of Medicine, Health and Sports, European University of Madrid, Villaviciosa de Odón, 28670 Madrid, Spain; isabel.minguez@universidadeuropea.es (I.M.-E.); angel.gonzalez@universidadeuropea.es (Á.G.-d.-l.-F.); mail@villafane.it (J.H.V.); 2 Department of Radiology, Rehabilitation and Physiotherapy, Faculty of Nursery, Physiotherapy and Podiatry, Complutense University of Madrid, 28040 Madrid, Spain; juavaler@ucm.es (J.A.V.-C.); gusplaza@ucm.es (G.P.-M.); 3 Grupo InPhysio, Instituto de Investigación Sanitaria del Hospital Clínico San Carlos (IdISSC), 28040 Madrid, Spain; 4 Department of Physical Therapy, Real Madrid C.F., 28055 Madrid, Spain; pebelon@gmail.com

**Keywords:** sciatic nerve, ultrasound, regression model, anthropometric data

## Abstract

**Objectives:** We aimed to create a predictive model to estimate sciatic nerve depth using anthropometric and demographic data to enhance safety and precession in needle-based interventions. Setting: The study was conducted at Universidad Europea de Madrid, Spain. **Methods:** A Cross-sectional observational study was carried out between January and April 2024. The study included fifty volunteers aged 18–45 years, without any muscle tone affections, lower limb asymmetries, or history of lower limb surgeries. Demographic and anthropometric data were collected, including sex, age, height, weight, BMI, and leg length measure and thigh circumference at specific points. The sciatic nerve depth was measured using ultrasound imaging under the gluteal fold and in the posterior middle third of the thigh. **Results:** Correlation analysis revealed significant associations between thigh circumference at the proximal and middle third and sciatic nerve depth. A multiple linear regression model identified that the proximal thigh circumference was a significant predictor of sciatic nerve depth, explaining 44.5% of the variance. The variance increased to 49.7% when gender was added. The depth of the sciatic nerve in the middle third explained 38.2% of the variance. And the inclusion of gender in the model explained 40.8% of the variance for the middle third. **Conclusions:** This study identify significant predictors such as the thigh girth at the proximal and mid-third levels, gender, and the BMI. These findings suggest that clinicians can use these anthropometric measurements to estimate sciatic nerve depth more accurately, reducing the risk of accidental nerve injury and improve the precision and safety of needling procedures during invasive procedures.

## 1. Introduction

The sciatic nerve (SN), which arises from the sacral plexus, specifically from the nerve roots L4-S3, usually exits the pelvic cavity through the great sciatic foramen [1]. It is recognized as the longest nerve in the human body. Its primary function is to innervate the posterior thigh muscles, continuing its course laterally to the ischial tuberosity [1,2]. Within the popliteal fossa, the nerve bifurcates into the tibial nerve and the common peroneal nerve; however, the precise location of bifurcation can vary due to individual anatomical differences [1]. Following this division, it passes through the popliteal fossa and then travels deeply between the soleus and gastrocnemius muscles [3].

Beaton and Anson [4] described six different relationships between the sciatic nerve and the piriformis muscle, the site of usual entrapment and consequent symptomatology. The type A pattern in which the SN passes through the piriformis muscle has the highest prevalence according to the systematic review by Poutoglidou F et al. [5].

Knowledge of the depth of the sciatic nerve is necessary when performing procedures such as nerve blocks. In 2006, a study was conducted with the main objective of estimating the depth of the sciatic nerve during a subgluteal block. A revision of 100 magnetic resonance images of the area was used to measure the thigh diameter, and after that, it was measured in 40 subjects undergoing a subgluteal sciatic block [6]. Bruhn et al. [7] mention in their article that the biceps femoris tendon can be a very useful tool for locating the sciatic nerve in the infragluteal region at an average distance of 2.6 ± 0.9 cm distal to the gluteal crease [7].

The application of invasive therapies such as dry needling, neuromodulation and electrolysis is currently on the rise [8,9]. Despite the safety and efficacy of these techniques, the use of a needle can lead to minor adverse effects such as bruising and pain during and after the procedure [10]. However, it is possible to generate more serious adverse effects in ≤0.04% of cases [11]. In any case, the risk of damage to nervous and vascular tissue remains a concern [12].

It is important to establish protocols that minimize the risk of injury since any technique involving the use of needles carries certain risks [13]. Measures to ensure the safety of both the patient and the physical therapist include hand washing, the disinfection of the area with chlorhexidine, the use of non-sterile gloves and the use of a single individualized sterile needle. Likewise, the correct positioning of the patient during the procedure, adapted according to the muscle being treated, is essential to reduce risks [14].

Moreover, it should be mentioned that ultrasound (US) has become an indispensable tool for the correct treatment and management of patients, particularly if the target is complex neuroanatomical structures such as the sciatic and tibial nerves [15]. Some of the advantages of the use of US are the minimization of risks and identification of structures and possible anatomical variations [16]. 

Ferrer-Peña et al. [16] have developed a prediction model based on anthropometric measurements based on the length and circumference of certain body areas, in combination with modulatory variables such as age, sex, height, weight and body mass index (BMI), to determine the length of the needle and consequently minimize the risk of nerve puncture [17].

The lack of recent scientific studies on sciatic nerve depth and the fact that the study performed by Crabtree E.C. et al. [6] is dated 2006 leads the present study to propose an assessment of sciatic nerve depth using ultrasound by using the new prediction model based on anthropometric measurements, such as the model developed by Valera-Calero et al. [18].

For this reason, the main objective of this study was to investigate whether it is possible to identify and predict sciatic nerve depth at two locations, either proximally or distally, through the anthropometric properties of age, weight, sex, height, the BMI and the circumference of the area to be assessed by using US. The alternative hypothesis of this study suggests that a linear regression model can identify statistically significant predictors of sciatic nerve depth in healthy subjects.

## 2. Methods

### 2.1. Study Design

A cross-sectional study was conducted to investigate potential predictors of sciatic nerve depth by analyzing the correlation between depth measurements at two anatomical locations and a range of demographic and anthropometric variables. A linear regression model was developed to help clinicians select the optimal needle length for non-ultrasound-guided needle interventions. This study followed the guidelines and checklist of the Strengthening the Reporting of Observational Studies in Epidemiology (STROBE) for cross-sectional studies. The Universidad Europea de Madrid Committee (CI/2024-507) supervised and approved all procedures and guaranteed the protection of participants’ rights in accordance with the Declaration of Helsinki.

### 2.2. Participants

Participants for the study were recruited at a private university in Madrid (Universidad Europea de Madrid) through local advertisements and online surveys from January 2024 to April 2024. Inclusion criteria included being aged between 18 and 45 years old, enabling the inclusion of a diverse demographic sample encompassing both genders, which may exhibit variations in sciatic nerve depth. 

Exclusion criteria included any pharmacological or physiotherapeutic treatment that could affect muscle tone; significant asymmetries in the lower extremities; a history of surgery in the lower extremities or in the lumbopelvic region; neuromuscular conditions affecting normal lumbopelvic morphology (e.g., sarcopenia; myasthenia gravis; amyotrophic lateral sclerosis; normal lumbopelvic muscle atrophy; or spinal muscular atrophy); pregnant women; or other possible medical conditions (e.g., tumor or fractures). All participants were required to sign a written informed consent form to participate in the study.

### 2.3. Sample Size Calculation

The recommendation of Beneciuk et al. [17] was taken as a reference, which mentions including 10 to 15 participants for each potential predictor and limiting the number of predictors to 5 to avoid overestimating [19] the precision, obtaining a minimum sample size of 50 participants. 

### 2.4. Demographic and Anthropometric Data

The following demographic data were included: sex (male/female), age (years), height (m), weight (kg) and the BMI (weight/height^2^) [20].

A tape measure was used to record the length of both legs, measured from the anterosuperior iliac spine to the external malleolus. Additionally, circumferences were taken at specific anatomical landmarks, including the lower gluteal fold and the posterior mid-third of the thigh.

### 2.5. Ultrasound Imaging Procedure: Sciatic Nerve

Participants were positioned in the prone decubitus position with their legs fully extended. All measurements were performed by a single examiner with over 5 years of experience in musculoskeletal ultrasound using a Sakura P10 ultrasound system (SonoScape, Shenzhen, China) equipped with a 12L-B linear transducer. US settings, including frequency, depth, focus and dynamic range, were individually optimized to ensure maximum image quality. To minimize measurement bias, only a slight pressure was applied to prevent the compression of the subcutaneous tissue.

### 2.6. Proximal Measurement1

**Figure 1:**
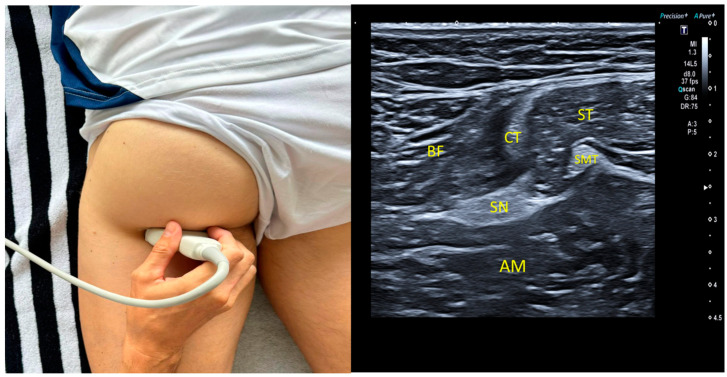
SN: sciatic nerve; CT: conjoint tendon; BF: biceps femoris; ST: semitendinous; SMT: semimembranosus tendon; AM: adductor magnus.

The procedure for imaging the sciatic nerve in the subgluteal region requires the subject to be in a prone position. The linear transducer needs to be placed under the gluteal fold, with the sciatic nerve located laterally to semitendinosus and semimembranosus muscles and medially to and deeply in the biceps femoris. As shown in Figure 1, US imaging allows a clear identification of the sciatic nerve relative to the surrounding anatomical structures. Perpendicular calipers can be employed to measure the distance (in centimeters) between the skin and the tibial nerve, ensuring accurate localization and assessment [1,6,21].

### 2.7. Mid-Third Measurement

**Figure 2:**
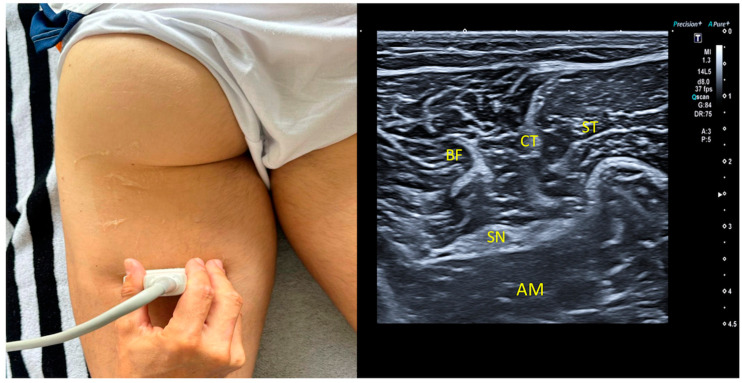
SN: sciatic nerve; CT: conjoint tendon; BF: biceps femoris; ST: semitendinous; SMT: semimembranosus tendon; AM: adductor magnus.

For the mid-third measurement, the transducer was placed at the midpoint of the posterior thigh. The muscular structures visualized include both heads of the biceps femoris. As shown in Figure 2, calipers were used to measure the distance from the skin to the sciatic nerve, ensuring the precise localization and assessment of the nerve at this location [1,7,21].

### 2.8. Statistical Analysis

The analysis procedure was performed with Jamovi Sofwtare (version 1.6, The Jamovi Project). Normal distribution was checked by the Saphiro–Wilk test (normal if *p* > 0.05). Student’s *t* test for independent samples was employed for analyzing gender and side differences. A Pearson’s correlation matrix was calculated to establish the association between normal distributed variables. Regarding the results, the interpretation correlation coefficients (r) were as follows: poor (r < 0.3), fair (0.3 < r < 0.5), moderate (0.6 < r < 0.8), or strong (r < 0.8). Finally, the variables that were reported to be statistically significant correlations (*p* < 0.05) with sciatic nerve depth were included in a forward stepwise multiple regression model to estimate the ultrasound distance. The significance threshold for including variables in the multiple linear regression model was determined by critical F values if they were reported to be *p* < 0.05. R^2^ adjusted values were reported step by step in the hierarchical regression model to determine the association of each additional variable.

## 3. Results

### 3.1. Sociodemographic Features

This study involved 50 participants, consisting of 27 males and 23 females. The mean age for the total sample was 23.2 years. Males had a mean age of 22.96 years, while females had a mean age of 23.57 years. There were significant differences between genders in terms of weight and height (*p* < 0.05). Males had a mean weight of 78.52 kg and a mean height of 1.79 m, whereas females had a mean weight of 64.96 kg and a mean height of 1.67 m. The overall BMI for the sample was 23.9 kg/m^2^, with males having a BMI of 24.47 kg/m^2^ and females a BMI of 23.32 kg/m^2^ (Table 1).

### 3.2. Anthropometric and Ultrasonography Characteristics

The anthropometric measurements and ultrasonographic features of the participants are detailed in Table 2. Males had a significantly greater leg length than females (95.92 ± 3.7 cm vs. 90.8 ± 5.8 cm (*p* < 0.001). However, there were no significant differences between genders in thigh girth measurements at both the proximal and mid-third (*p* > 0.05). Additionally, males had a significantly more superficial sciatic nerve depth at the proximal third (3.3 ± 0.4 mm vs. 3.8 ± 0.7 mm, *p* = 0.001). There were no significant differences in sciatic nerve depth at the mid-third or between the left and right legs for any of the measurements (*p* > 0.05).

### 3.3. Correlation Analysis

The Pearson correlation coefficients, as shown in Table 3, reveal significant associations between various anthropometric features and the depth of the sciatic nerve. Thigh girth at both the proximal and mid-third levels showed a strong positive correlation with sciatic nerve depth at the corresponding levels (r = 0.671 to 0.703, *p* < 0.001). The BMI also showed a significant positive correlation with sciatic nerve depth at both the proximal (r = 0.534, *p* < 0.001) and mid-third (r = 0.614, *p* < 0.001). Leg length and height were not significantly correlated with sciatic nerve depth. These results highlight the importance of thigh girth and the BMI as predictors of sciatic nerve depth.

### 3.4. Hierarchical Regression Analysis

Table 4 summarizes the predictors of sciatic nerve depth at both the proximal and mid-third locations. Regarding the proximal sciatic nerve depth, thigh girth at the proximal third was a significant predictor of sciatic nerve depth, explaining 44.5% of the variance. In step 2, adding the gender variable, the explained variance increased to 49.7%. Considering the mid-third sciatic nerve depth, step 1 showed that the mid-third girth was a significant predictor, explaining 38.2% of the variance. Gender in the model explained 40.8% of the variance. Last, thigh girth at the proximal third, gender, weight and thigh girth at the mid-third explained 45.9% of the variance. Therefore, thigh girth at the proximal third and gender were significant predictors, while weight and thigh at the mid-third were not significant.

### 3.5. Proximal Sciatic Nerve Depth Prediction

**Figure 3:**
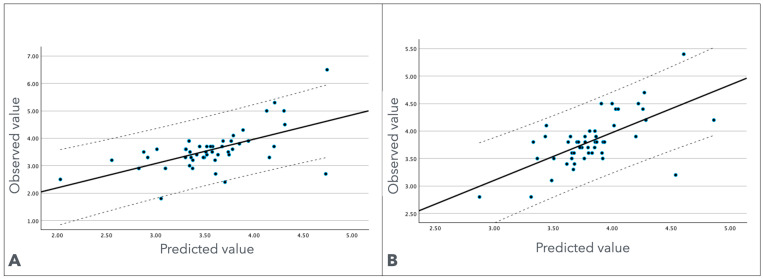
Scatter plot showcasing the relationship between observed values (Y-axis) and predicted values (X-axis) for the distance from the skin surface to the sciatic nerve (crossmarks, measured in cm) at the proximal third (**A**) and mid-third (**B**). The solid black line depicts the linear regression model, while the dashed lines represent the 95% prediction interval boundaries.

Based on the hierarchical regression analysis presented in Table 4, a practical formula was developed to select the appropriate needle length for the dry needling of the proximal sciatic nerve (Figure 3A). The regression equation derived from the results is as follows:

Depth = β0 + (β1 × Gender) + (β2 × Thigh Girth at Proximal Third)

Table 4 shows that the regression coefficients are as follows: intercept (β0): 0.091; gender (β1): −0.316; and thigh girth at the proximal third (β2): 0.088.

Thus, the formula to predict proximal sciatic nerve depth is as follows:

Predicted Proximal Sciatic Nerve Depth (mm) = 0.091 + (−0.316 × Gender) + (0.088 × Thigh Girth at Proximal Third).

### 3.6. Mid-Third Sciatic Nerve Depth Prediction

Similarly, a practical formula was developed to select the appropriate needle length for the dry needling of the mid-third sciatic nerve. This formula incorporates significant predictors identified in the study: gender, weight, thigh girth at the mid-third, and thigh girth at the proximal third (Figure 3B). The regression equation derived from the results is as follows:

Depth = β0 + (β1 × Gender) + (β2 × Weight) + (β3 × Thigh Girth at Midthird) + (β4 × Thigh Girth at Proximal Third)

Table 4 shows that the regression coefficients are as follows: intercept (β0): 0.060; gender (β1): −0.109; weight (β2): 0.004; thigh girth at the mid-third (β3): 0.018; and thigh girth at the proximal third (β4): 0.001.

Thus, the formula to predict mid-third sciatic nerve depth is as follows:

Predicted Midthird Sciatic Nerve Depth (mm) = 0.060 + (−0.109 × Gender) + (0.004 × Weight) + (0.018 × Thigh Girth at Mid-third) + (0.001 × Thigh Girth at Proximal Third).

## 4. Discussion

The authors consider this to be the first study conducted with the purpose of assisting clinicians in preventing accidental punctures during invasive sciatic nerve treatment techniques. The results revealed several easily measurable factors associated with sciatic nerve depth, such as gender, the BMI and thigh circumference.

The correlation analysis revealed significant associations between thigh circumference at the proximal and middle third s and sciatic nerve depth. Similarly, the BMI showed significant associations at both locations. Proximal thigh circumference was a significant predictor of sciatic nerve depth, explaining 44.5% of the variance. The variance increased to 49.7% when gender was added. Regarding the middle third, the depth of the sciatic nerve in the middle third explained 38.2% of the variance. And the inclusion of gender in the model explained 40.8% of the variance. Finally, the inclusion of thigh circumference in the proximal third, gender, weight and thigh circumference in the middle third explained 45.9% of the variance. Notably, thigh circumference in the proximal third and sex were significant predictors, while weight and thigh circumference in the middle third were not.

As in recent publications, the results of this article highlight the possibility of using anthropometric measurements to predict the depth of neurovascular structures. There are several articles in which Valera Calero et al. have studied the possible correlation between anthropometric measurements and needle length selection to ensure safe dry needling practices [18,22,23]. For example, they have identified that gender, the BMI, and chest circumference could predict both rhomboid muscle and pleural depth [20]. In addition, the correct localization of the needles is essential for the treatments whose main objective is the modulation of the nerve in addressing the different pathologies in which it may be affected [23]. 

It is remarkable to mention that the BMI showed a significant and positive correlation regarding the depth of the sciatic nerve both proximally and in the middle third; this underlines the importance of knowing the BMI. It is decisive since a higher BMI is associated with a higher percentage of fat and consequently with a higher layer of adipose tissue, and therefore, the vascular and nervous structures will be found at deeper levels [24].

However, Crabtree E.C. et al. [6] point out in their article that the sciatic nerve is located at 43% of the diameter of the thigh in the lateral position [6]. It should be borne in mind that for invasive techniques in physiotherapy, the position for the sciatic nerve approach is in supine decubitus, and so, for this reason, it is equally important to establish the depth of the sciatic nerve in this position [25]. Likewise, using the formula presented, the size of the needle can be calculated to prevent the risk of accidental punctures of vascular and nervous tissues. For this reason, the present study was conducted.

In addition, the results of this study are useful for the correct treatment of myofascial pathology such as trigger points by the dry needling of the biceps femoris, semitendinosus and semimembranosus muscles [26]. Through the application of the derived formulas, clinicians can ensure that the needles achieve the appropriate depth, increasing the efficiency of the treatment and reducing the potential risks.

Furthermore, it should be mentioned that the present formulas can be used to treat one of the most common pathologies in the lower limb, sciatic nerve neuropathy. This could be due to an acute cause such as a hip fracture or hip arthroplasty, or it could be due to a maintained compression, as well as nerve entrapment [27]. Regarding the physiotherapeutic approach, there is an invasive technique known as percutaneous neuromodulation (PMN) guided by ultrasound [28]. It consists of applying percutaneous electrical stimulation through a needle-shaped electrode placed close to the nerve [28]. For instance, tibial nerve neuromodulation treatment has been shown to have significant efficacy in the treatment of urinary incontinence [29]. These results can be very valuable for optimizing therapeutic interventions in musculoskeletal conditions of the aforementioned area, muscles irradiated by the sciatic nerve and even for the sciatic nerve approach.

## 5. Limitations

Despite the relevant information described in the article, several limitations should be considered. Firstly, there was a possible limitation regarding the sociodemographic and anthropometric characteristics of the participants. Similarly, another limitation is the subject profile, which should be considered to include individuals with diverse clinical conditions and those with anatomical variations to improve the accuracy and generalizability of the model. Another limitation of the study is the anatomical variability as well as the different echogenicity of the subjects. It is important to address these limitations in future research to strengthen the findings of this study by achieving more reliable and effective methods for predicting sciatic nerve depth. This will also improve the clinical outcomes of the different treatments.

## 6. Conclusions

This study identified significant predictors of sciatic nerve depth at both the proximal and mid-third locations of the thigh. Thigh girth at the proximal and mid-third levels, gender, and the BMI emerged as key predictors. For the proximal sciatic nerve depth, gender and thigh girth at the proximal third explained 47.9% of the variance. For the mid-third sciatic nerve depth, thigh girth at the proximal third, gender, weight, and thigh girth at the mid-third explained 45.9% of the variance. These findings suggest that clinicians can use these anthropometric measurements to estimate sciatic nerve depth more accurately, reducing the risk of accidental nerve injury and improve the precision and safety of needling procedures during invasive procedures.

## Figures and Tables

**Table 1:** Sociodemographic data of the participants.

Variables	Total Sample (*n* = 50)	Males (*n* = 27)	Females (*n* = 23)
Age, y	23.2 ± 3.46	22.96 ± 3.20	23.57 ± 3.78
Weight, kg †	72.3 ± 12.1	78.52 ± 7.31	64.96 ± 12.53
Height, m †	1.73 ± 0.08	1.79 ± 0.04	1.67 ± 0.06
BMI, kg/m^2^	23.9 ± 3.29	24.47 ± 2.14	23.32 ± 4.23

† Significant between-gender differences (*p* < 0.05).

**Table 2:** Anthropometric and ultrasonographic features by gender and leg side.

	Gender				Leg Side	
Variables	Males (*n* = 27)	Females (*n* = 23)	Difference	Left (*n* = 50)	Right (*n* = 50)	Difference
Leg Length (cm)	95.92 ± 3.7	90.8 ± 5.8	−5.0 (−7.0; −3.1) *p* < 0.001	93.5 ± 5.4	93.7 ± 5.4	−0.2 (−2.4; 1.9) *p* = 0.83
Thigh Girth (cm)—Proximal Third	60.5 ± 3.8	61.6 ± 5.8	1.1 (−1.5; 1.8) *p* = 0.86	61.0 ± 4.8	60.9 ± 4.9	0.1 (−1.8; 1.9) *p* = 0.94
Thigh Girth (cm)—Mid-Third	54.0 ± 3.7	53.5 ± 4.6	0.1 (−0.8; 3.0) *p* = 0.25	54.1 ± 4.0	54.0 ± 4.3	0.0 (−1.5; 1.7) *p* = 0.97
Sciatic Nerve Depth (mm)—Proximal Third	3.3 ± 0.4	3.8 ± 0.7	0.4 (0.1; 0.6) *p* = 0.001	3.5 ± 0.7	3.5 ± 0.5	0.0 (−0.2; 0.2) *p* = 0.97
Sciatic Nerve Depth (mm)—Mid-Third	3.8 ± 0.3	3.9 ± 0.5	4.3 (0.0; 0.3) *p* = 0.126	3.8 ± 0.4	3.9 ± 60.4	0.0 (−0.2; 0.2) *p* = 0.40

Baseline values are expressed as mean ± standard deviation; between-groups differences are expressed as mean (95% confidence interval) and *p*-values.

**Table 3:** Pearson product moment correlation matrix.

	1	2	3	4	5	6	7	8	9
1. Leg length	—								
2. Thigh girth proximal	−0.033	—							
3. Thigh girth mid-third	−0.154	0.876 ^†^	—						
4. Age	−0.154	−0.126	−0.096	—					
5. Weight	0.324 *	0.602 ^†^	0.590 ^†^	−0.189	—				
6. Height	0.688 ^†^	−0.009	−0.053	−0.302 *	0.621 ^†^	—			
7. BMI	−0.081	0.764 ^†^	0.779 ^†^	−0.036	0.826 ^†^	0.075	—		
8. SN depth proximal	−0.148	0.671 ^†^	0.571 ^†^	−0.104	0.262	−0.270	0.534 ^†^	—	
9. SN depth mid-third	−0.012	0.703 ^†^	0.623 ^†^	−0.220	0.418 ^†^	−0.113	0.614 ^†^	0.755 ^†^	—

Note: * *p* < 0.05, ^†^ *p* < 0.001. Abbreviations: SN, sciatic nerve.

**Table 4:** Hierarchical regression model to determine predictors sciatic nerve depth at proximal and mid-third depth.

	Predictor Outcome	B	SE B	95% CI	β	t	*p*
Proximal	Step 1						
	Thigh girth proximal	0.091	0.01	(0.523, 0.820)	0.671	8.96	<0.001
	Step 2						
	Gender	−0.316	0.094	(−0.764, −0.195)	−0.479	−3.35	0.001
	Thigh girth proximal	0.088	0.00	(0.501, 0.786)	0.643	8.97	<0.001
Mid-third	Step 1						
	Thigh girth mid-third	0.067	0.00	(0.466, 0.780)	0.623	7.886	<0.001
	Step 2						
	Weight	0.003	0.005	(−0.203, 0.365)	0.081	0.574	0.571
	Thigh girth mid-third	0.066	0.016	(0.287, 0.856)	0.571	4.04	<0.001
	Step 3						
	Gender	−0.305	0.142	(−1.269, −0.042)	−0.656	−2.151	0.037
	Weight	0.014	0.007	(−0.016, 0.748)	0.366	1.928	0.060
	Thigh girth mid-third	0.018	0.018	(0.089, 0.720)	0.405	0.405	0.013
	Step 4						
	Thigh girth proximal	0.060	0.025	(0.080, 1.160)	0.620	2.313	0.025
	Gender	−0.109	0.260	(−0.926, 0.458)	−0.233	−0.680	0.500
	Weight	0.004	0.008	(−0.327, 0.535)	0.104	0.486	0.629
	Thigh girth mid-third	0.001	0.026	(−0.443, 0.468)	0.012	0.054	0.957

Proximal sciatic nerve depth: R^2^ adj. = 0.445 for step 1 (F = 80.3; *p* < 0.001), R^2^ adj. = 0.497 for step 2 (F = 50.0, *p* < 0.001). Mid-third sciatic nerve depth: R^2^ adj. = 0.382 for step 1 (F = 62.2, *p* < 0.001), R^2^ adj. = 0.362 for step 2 (F = 14.9, *p* < 0.001), R^2^ adj. = 0.408 for step 3 (F = 12.2, *p* < 0.001), R^2^ adj. = 0.459 for step 4 (F = 11.4, *p* < 0.001).

## Data Availability

Data can be retrieved via sending a formal request to the corresponding author.
